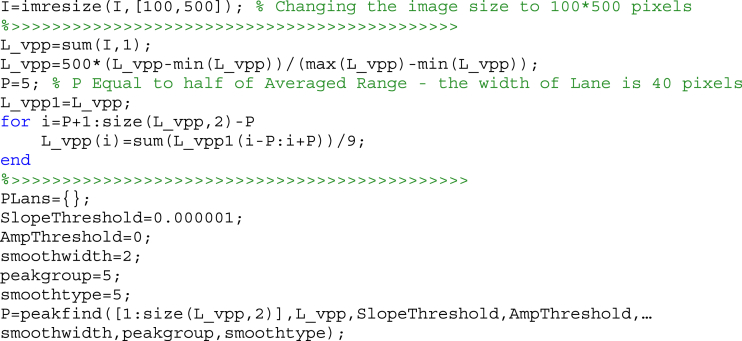# Code and data on the processing of the pulsed-field gel electrophoresis images: A matlab script

**DOI:** 10.1016/j.dib.2019.105035

**Published:** 2019-12-20

**Authors:** Ahmad Mohammadbeigi, Mohammad Rezaei, Naser Zohourian Sani, Nemat Soltani, Parviz Mohajeri

**Affiliations:** a Student Research Committee, Kermanshah University of Medical Sciences, Kermanshah, Iran; b Department of Radiology, Sina Hospital, Tehran University of Medical Sciences, Tehran, Iran; c Sleep Disorders Research Center, Kermanshah University of Medical Sciences, Kermanshah, Iran; d Department of Medical Physics and Biomedical Engineering, School of Medicine, Tehran University of Medical Sciences, Tehran, Iran; e Department of Microbiology, School of Medicine, Kermanshah University of Medical Sciences, Kermanshah, Iran; f Bio Medical Engineering Unit, Taleghani Hospital, Mashhad University of Medical Sciences, Mashhad, Iran

**Keywords:** Band detection, Pattern recognition, Pulsed-field gel electrophoresis, Image processing, Matlab script

## Abstract

Here a matlab script was presented for lane tracking and band detection on the pulsed field gel electrophoresis (PFGE) images. It can also be used as a software tool for automatic analysis of PFGE images. The data consist of several MATLAB codes which collectively have the task of lane tracking, band detecting and pattern recognition on the PFGE images. The lane tracking stage is semi-automatic and the band detection stage is fully automatic. Finally, the pattern of lanes that includes number of, location, width and light intensity level of bands was obtained.

**Table:** Specifications Table

Subject	Biomedical Engineering
Specific subject area	image processing in microbiology and biotechnology
Type of data	MATLAB code, image, video
How data were acquired	All source codes written in Matlab software.
Data format	MATLAB code, JPEG, Mp4
Parameters for data collection	All the codes were implemented in MATLAB-R2009a on a system with Intel Core - i5 2430M, quad-core processor overclocked at 3.2 GHz with 8GB of RAM clocked at 1600 MHz. A trial version of GelCompar II version 6.6.11 was used to evaluate and optimize the codes.
Description of data collection	The images were captured using PFGE BIORAD at the Microbiology Laboratory of Kermanshah University of Medical Sciences. The images were provided by two types of bacteria, including Acineto-AF, *Staphylococcus aureus*.
Data source location	Institution: Department of Biomedical Engineering in Kermanshah University of Medical Science City/Town/Region: Kermanshah Country: Iran Latitude and longitude: 34°23′27.9″N 47°06′07.7″E
Data accessibility	- with the article - *The dataset is freely available at* [1] *for any academic, educational, and research purposes.* Repository name: Mendeley Data Data identification number: https://doi.org/10.17632/mcnfncf25t.1 Direct URL to data: https://data.mendeley.com/datasets/mcnfncf25t/1
Related research article	Author's name: Mohammad Rezaei, Mahmood Amiri, Parviz Mohajeri, Mansour Rezaei Title: A new algorithm for lane detection and tracking on pulsed field gel electrophoresis images Journal: Chemometrics and Intelligent Laboratory Systems DOI: 10.1016/j.chemolab.2016.05.018

**Table:** 

**Value of the Data** • The provided codes can be used to pulsed-field gel electrophoresis image analysis. • The Matlab script will allow microbiologist to molecular subtyping. • This approach can be used to automatic lane tracking, band detection and pattern recognization on PFGE images.

## 1 Data

**Fig. 1:**
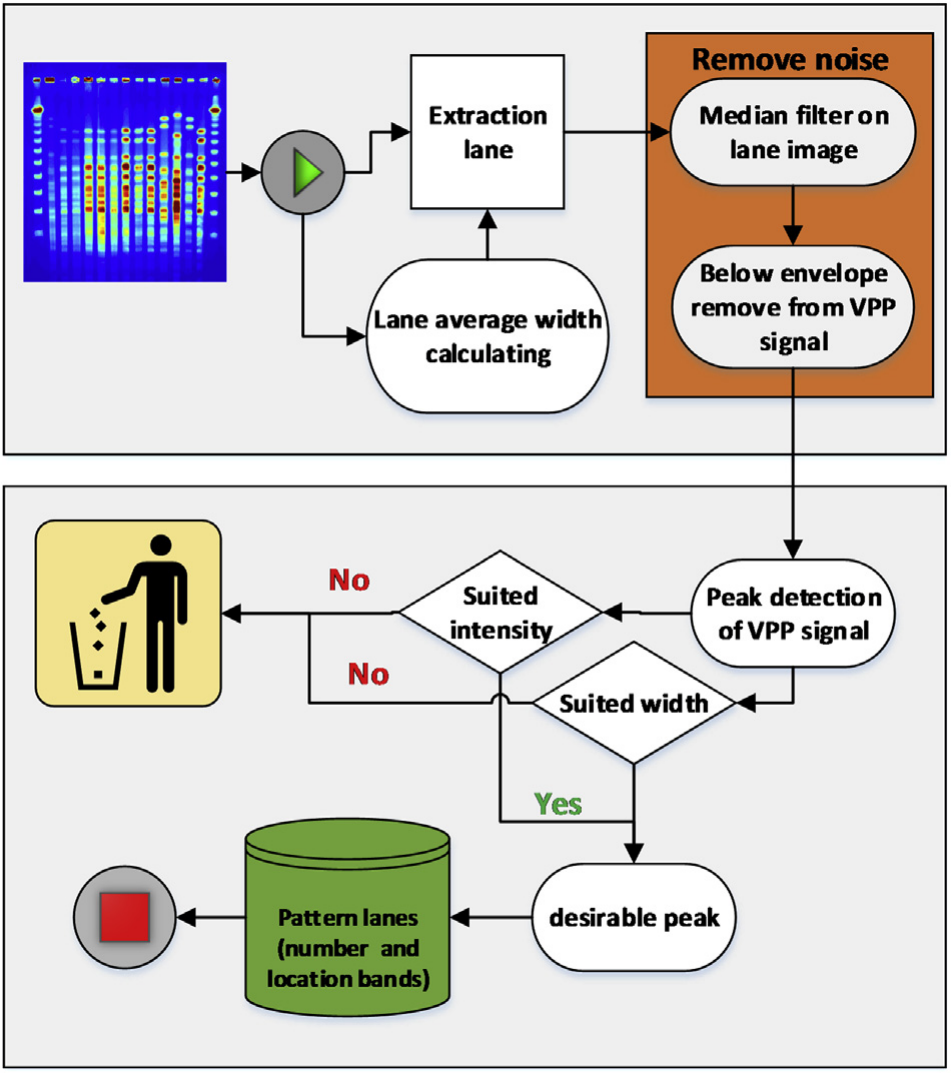
The flowchart of the proposed algorithm. It composed of three phases:1- Lane detection 2- Noise reduction and band extraction 3- Pattern recognition.

The data consist of several MATLAB codes, which collectively have the task of lane tracking, band detecting and pattern recognition on the pulsed-field gel electrophoresis images. The PFGE is a laboratory technique used by researchers and scientists to produce a DNA fingerprint for a bacterial isolate as a group of the same type of bacteria [[2], [3], [4], [5]]. The images were provided by two types of bacteria, including Acinetobacter [6], *Staphylococcus aureus* [7] which were attached with the article. All the codes and data needed for this purpose available in the Mendeley data source [1]. In addition, there is a video showing the performance of the data and Matlab script. The flowchart of implemented algorithm as Matlab script was shown in Fig. 1.

## 2 Experimental design, materials, and methods

The material used in this dataset includes images and codes. The images were collected using PFGE BIORAD at the Microbiology Laboratory of Kermanshah University of Medical Sciences in “*tiff*” format. To analyse the images, MATLAB-R2009a [8] on a system with Intel Core - i5 2430M, quad-core processor overclocked at 3.2 GHz with 8GB of RAM clocked at 1600 MHz was used. To evaluate and optimize the codes, a trial version of GelCompar II software was also used.

### 2.1 Algorithm for the lane tracking

**Fig. 2:**
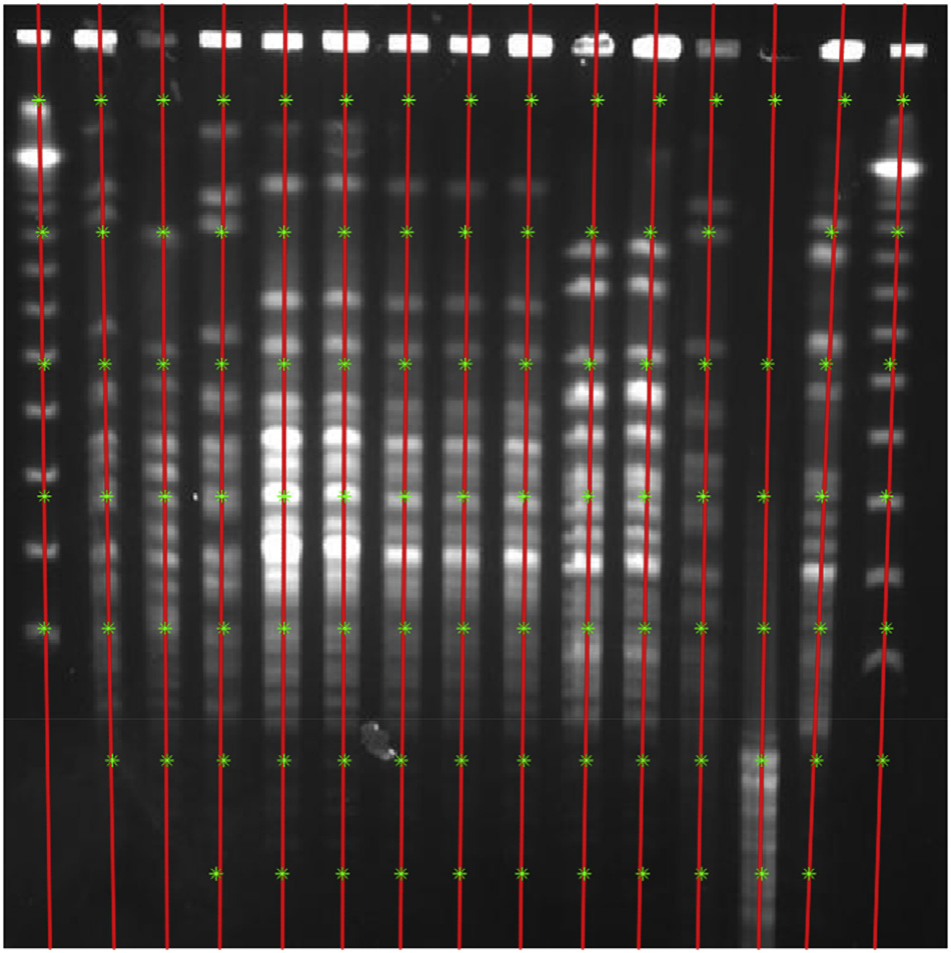
A PFGE image with lanes which tracked using the matlab script “*lane_tracking.m*”: The red lines demonstrate lanes and the green stars are center of the lanes.

A program, “*lane_tracking.m*”, is responsible for lane tracking. First, image was converted to gray-scale from RGB format. The desired area of the image including lanes was cropped then was resized to 500 × 500 pixels. Next, image segmented to sub-images to calculate vertical projection profile (VPP). To detect local of each lane in the sub-images, local maxima from signal of VPP was detected. Using the matlab codes, “*widthfind.m*” and “*Remov_locmax.m*”, false-positive local maxima in the background was removed. After detecting the center of lanes in each of sub-images, the centers associated with each lane are given to the function “*createFit.m*” to fit the patch of lane. In detail, the proposed algorithm is described in Ref. [9]. Fig. 2 depicts the lane tracking.

### 2.2 Algorithm for the band detection

The band detection as one of the stages consists of two phases. First, a matlab code, “*bandremovebackgroundm.m*”, is responsible for removing background noise from lanes image. To this end, the lane image was smoothed by an adaptive median filter on lane images. Then, as a completion step, lower envelope of VPP of lane was subtracted. The source code for this function is given bellow.

**Figure:**
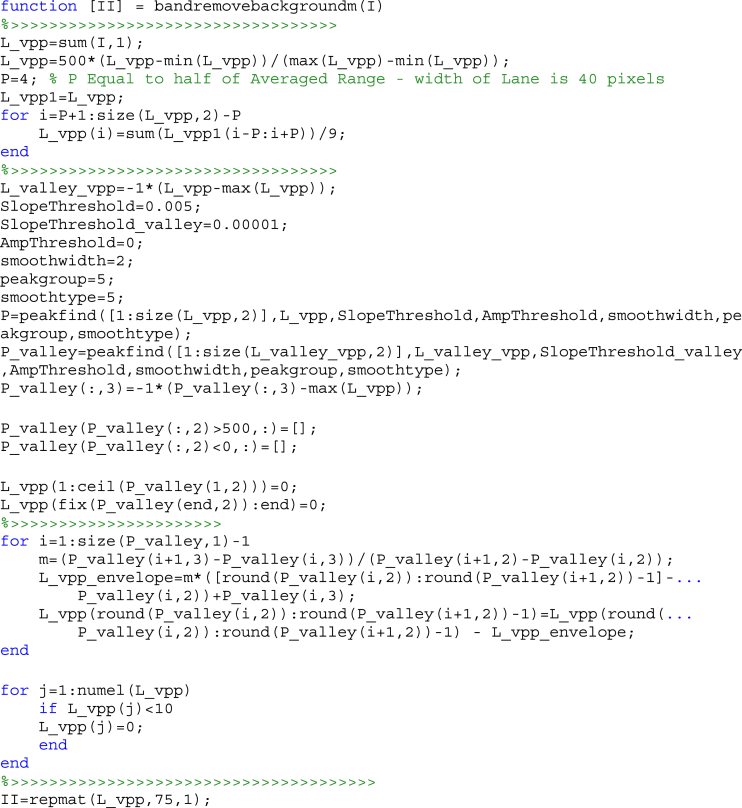

In addition, the program “*band.m*” is responsible for band detection. To this end, after resizing it to 100 × 500 pixels and calculating the VPP of lane, the location of the bands was detected using the function “peakfind.m”. The source code for this step is as follows. Finally, the pattern of lanes was determined by calculating the four parameters, including number of location, width and light intensity level of bands.

**Figure:**