# Supplementary figures and images for: Code and data on the processing of the pulsed-field gel electrophoresis images: A matlab script
Source: Data Brief. 2019 Dec 20;28:105035. doi: 10.1016/j.dib.2019.105035 (PMC6940661; doi:10.1016/j.dib.2019.105035)

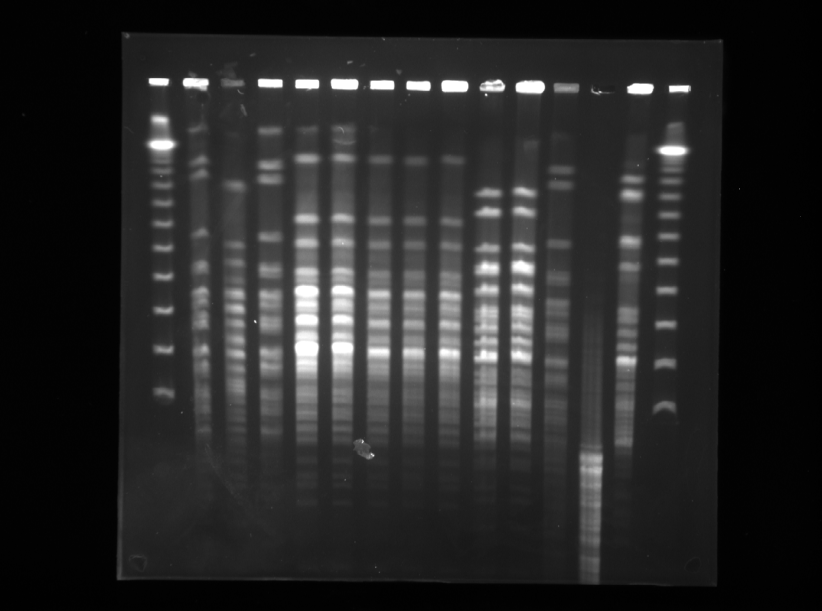

Supplement: Multimedia component 1 [file mmc1.zip › dib_105035_PFGE images for Acineto-AF/PFGE images for Acineto-AF/Acineto-AF (1).tif]

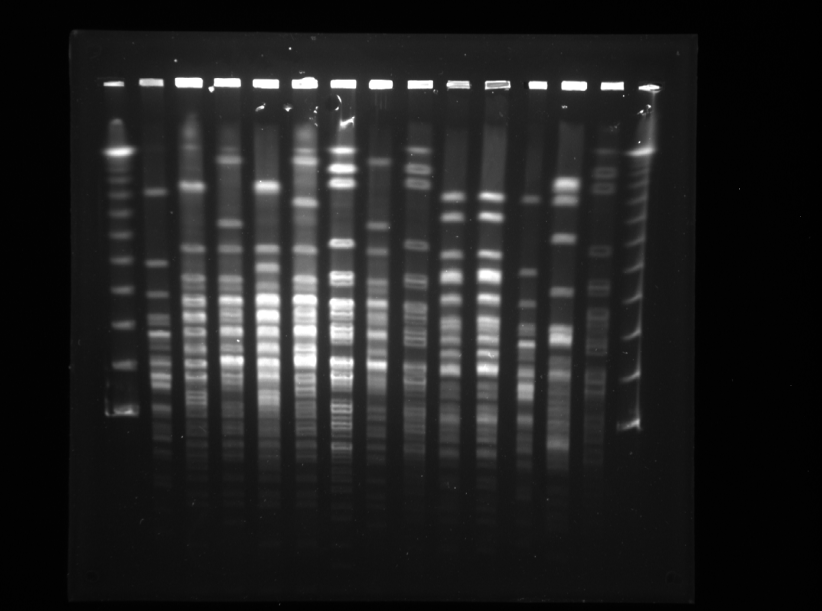

Supplement: Multimedia component 1 [file mmc1.zip › dib_105035_PFGE images for Acineto-AF/PFGE images for Acineto-AF/Acineto-AF (10).tif]

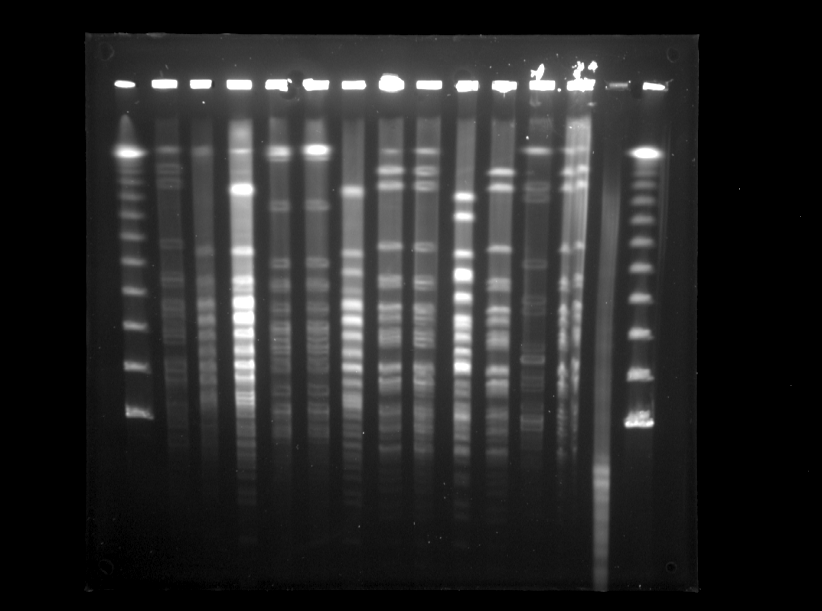

Supplement: Multimedia component 1 [file mmc1.zip › dib_105035_PFGE images for Acineto-AF/PFGE images for Acineto-AF/Acineto-AF (11).tif]

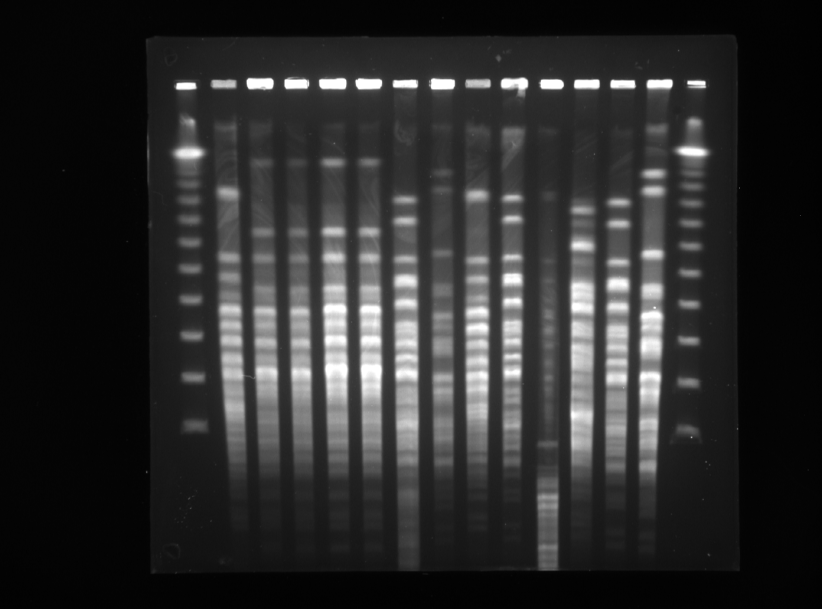

Supplement: Multimedia component 1 [file mmc1.zip › dib_105035_PFGE images for Acineto-AF/PFGE images for Acineto-AF/Acineto-AF (2).tif]

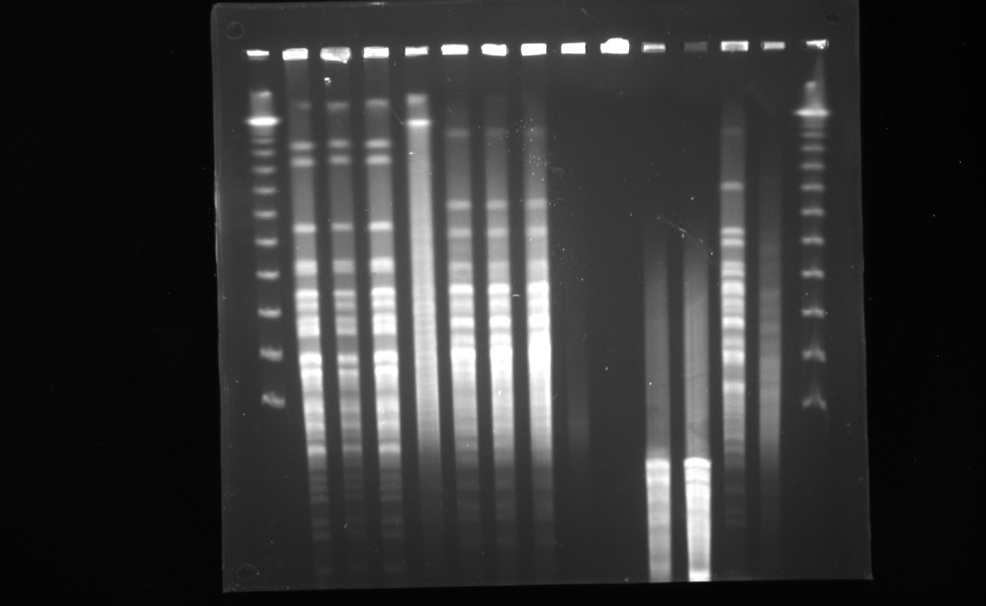

Supplement: Multimedia component 1 [file mmc1.zip › dib_105035_PFGE images for Acineto-AF/PFGE images for Acineto-AF/Acineto-AF (3).tif]

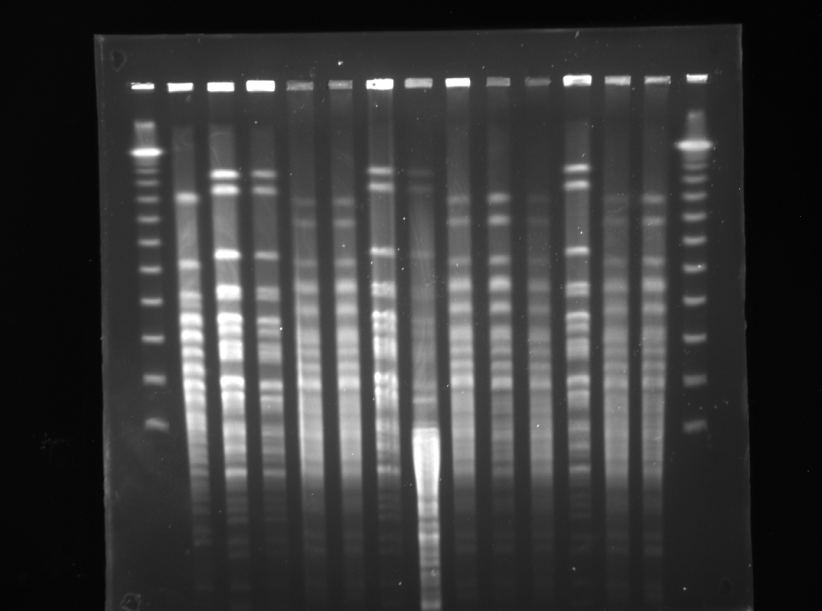

Supplement: Multimedia component 1 [file mmc1.zip › dib_105035_PFGE images for Acineto-AF/PFGE images for Acineto-AF/Acineto-AF (4).tif]

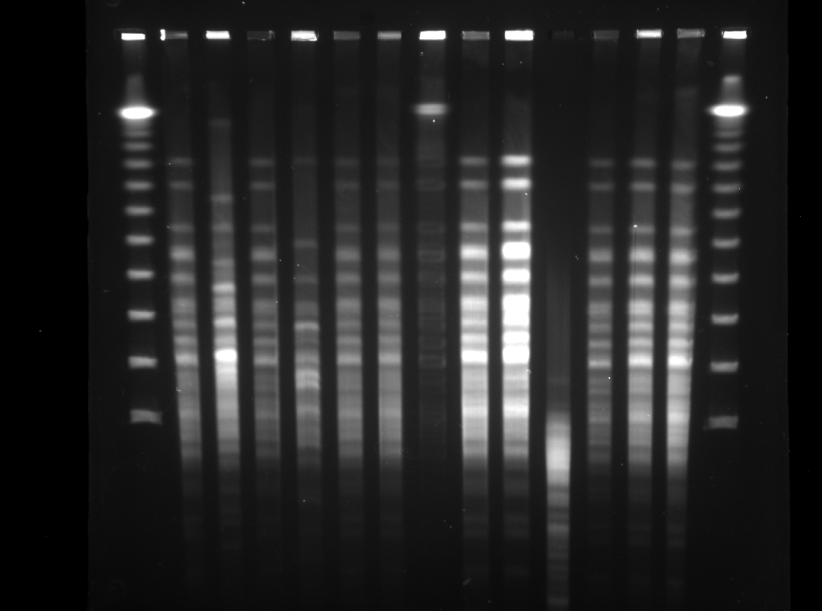

Supplement: Multimedia component 1 [file mmc1.zip › dib_105035_PFGE images for Acineto-AF/PFGE images for Acineto-AF/Acineto-AF (5).tif]

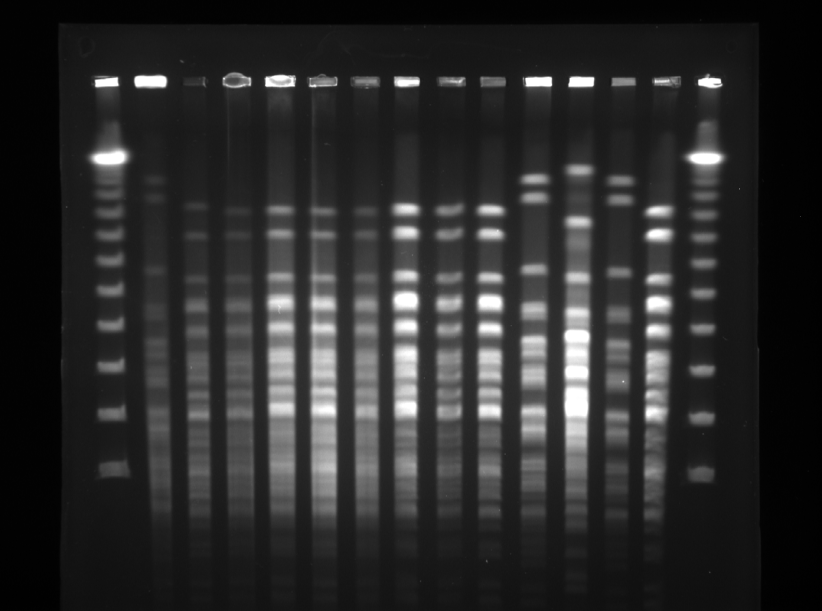

Supplement: Multimedia component 1 [file mmc1.zip › dib_105035_PFGE images for Acineto-AF/PFGE images for Acineto-AF/Acineto-AF (6).tif]

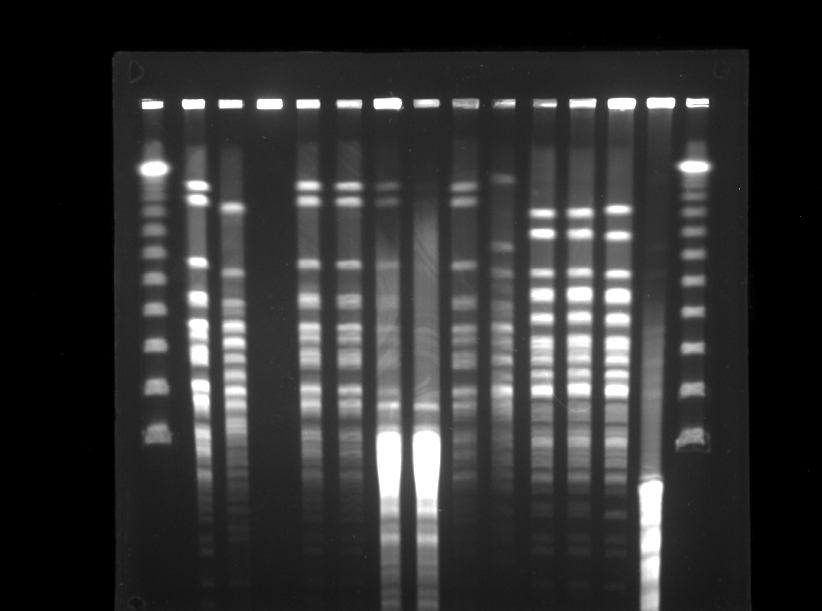

Supplement: Multimedia component 1 [file mmc1.zip › dib_105035_PFGE images for Acineto-AF/PFGE images for Acineto-AF/Acineto-AF (7).tif]

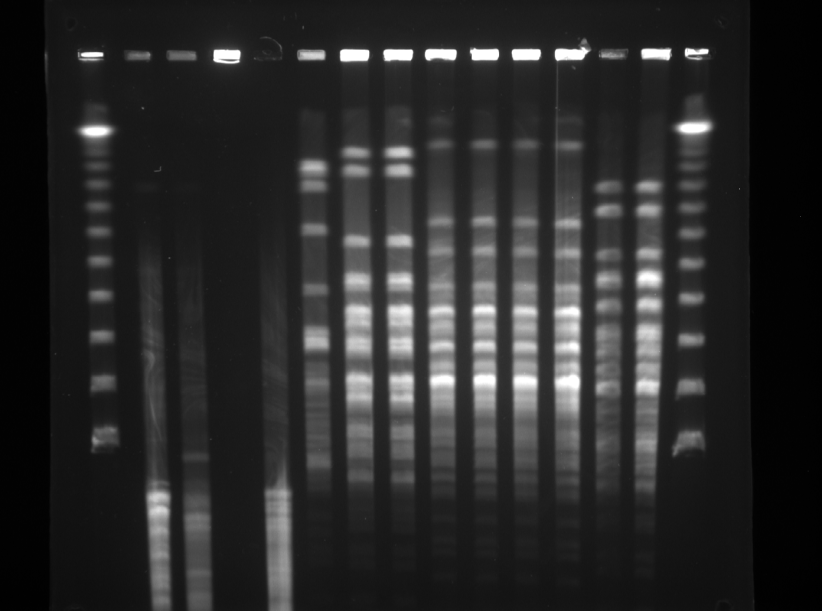

Supplement: Multimedia component 1 [file mmc1.zip › dib_105035_PFGE images for Acineto-AF/PFGE images for Acineto-AF/Acineto-AF (8).tif]

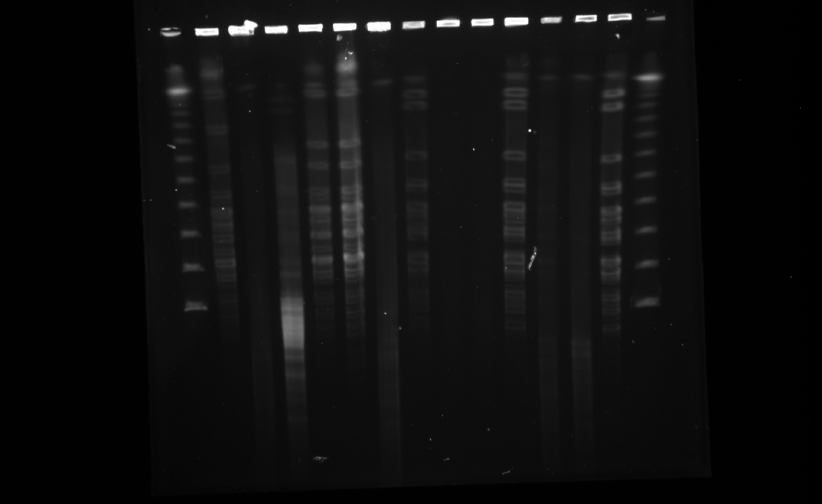

Supplement: Multimedia component 1 [file mmc1.zip › dib_105035_PFGE images for Acineto-AF/PFGE images for Acineto-AF/Acineto-AF (9).tif]

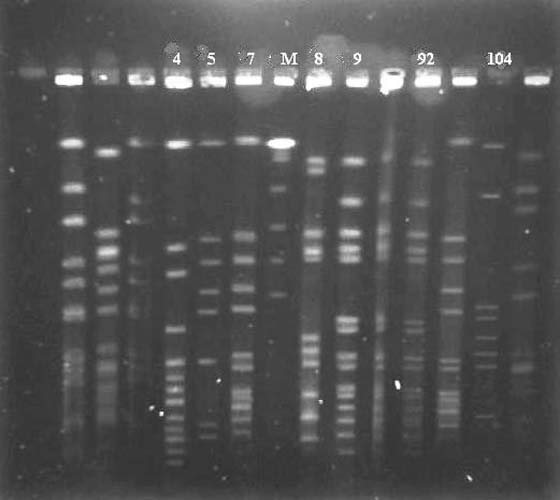

Supplement: Multimedia component 2 [file mmc2.zip › dib_105035_PFGE images for Staphylococcus Aureus/PFGE images for Staphylococcus Aureus/Staphylococcus aureus (1).tif]

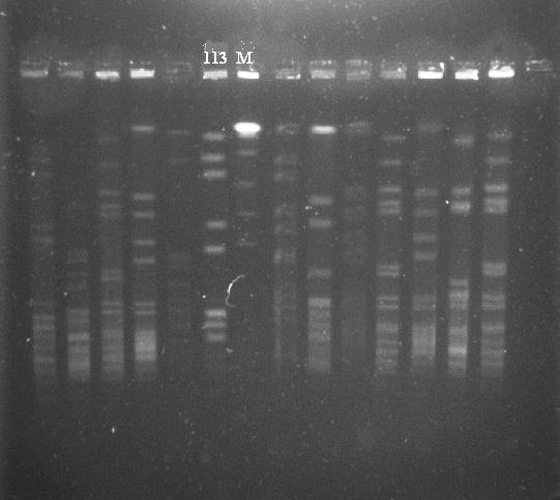

Supplement: Multimedia component 2 [file mmc2.zip › dib_105035_PFGE images for Staphylococcus Aureus/PFGE images for Staphylococcus Aureus/Staphylococcus aureus (10).tif]

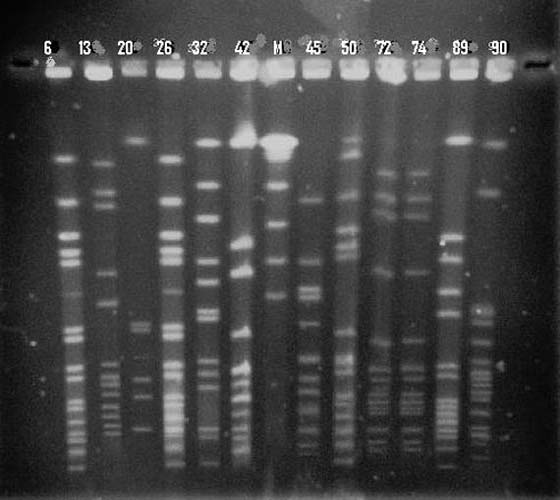

Supplement: Multimedia component 2 [file mmc2.zip › dib_105035_PFGE images for Staphylococcus Aureus/PFGE images for Staphylococcus Aureus/Staphylococcus aureus (2).tif]

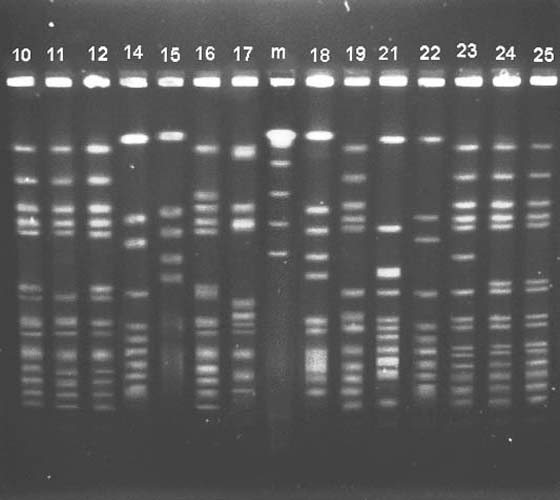

Supplement: Multimedia component 2 [file mmc2.zip › dib_105035_PFGE images for Staphylococcus Aureus/PFGE images for Staphylococcus Aureus/Staphylococcus aureus (3).tif]

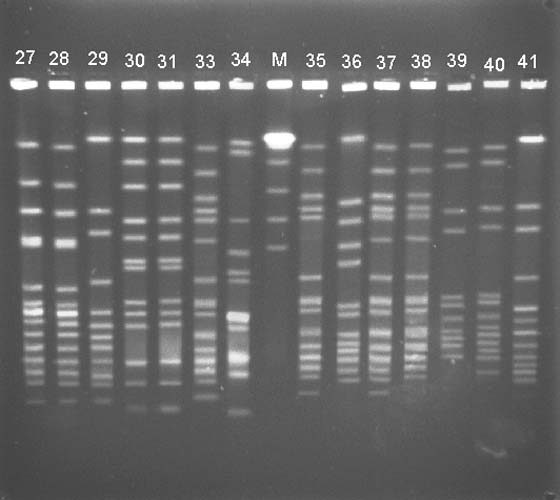

Supplement: Multimedia component 2 [file mmc2.zip › dib_105035_PFGE images for Staphylococcus Aureus/PFGE images for Staphylococcus Aureus/Staphylococcus aureus (4).tif]

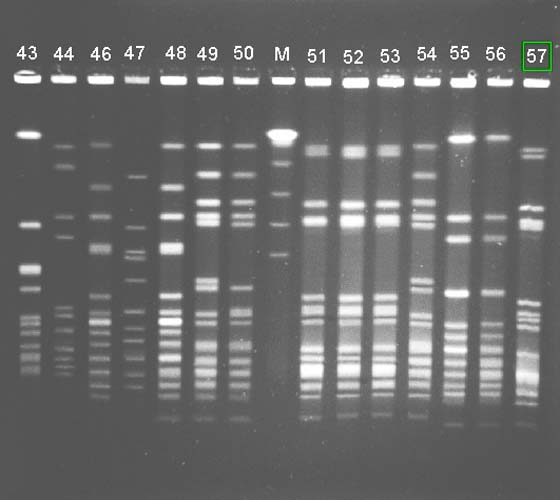

Supplement: Multimedia component 2 [file mmc2.zip › dib_105035_PFGE images for Staphylococcus Aureus/PFGE images for Staphylococcus Aureus/Staphylococcus aureus (5).tif]

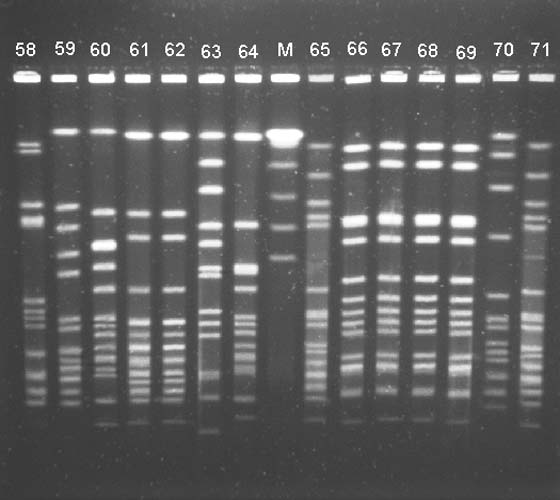

Supplement: Multimedia component 2 [file mmc2.zip › dib_105035_PFGE images for Staphylococcus Aureus/PFGE images for Staphylococcus Aureus/Staphylococcus aureus (6).tif]

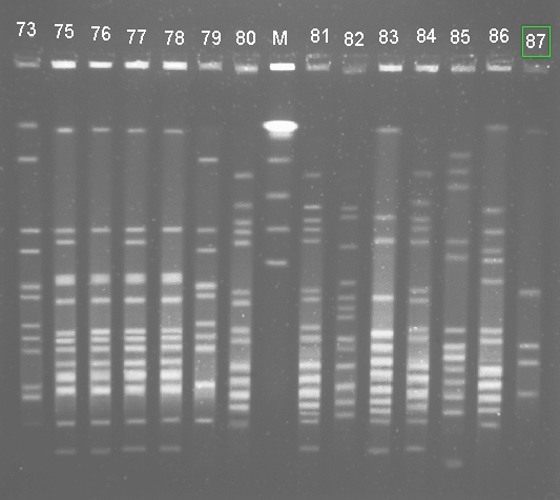

Supplement: Multimedia component 2 [file mmc2.zip › dib_105035_PFGE images for Staphylococcus Aureus/PFGE images for Staphylococcus Aureus/Staphylococcus aureus (7).tif]

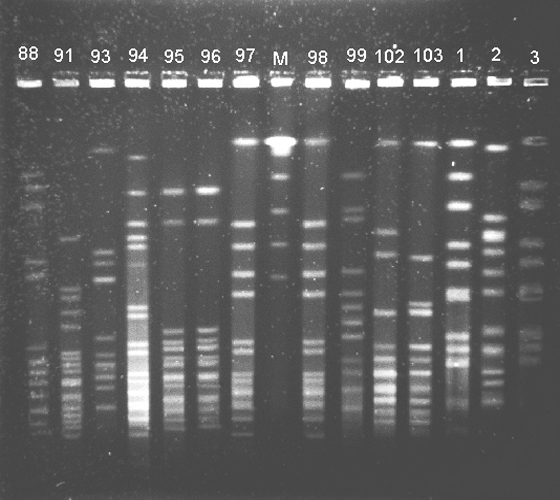

Supplement: Multimedia component 2 [file mmc2.zip › dib_105035_PFGE images for Staphylococcus Aureus/PFGE images for Staphylococcus Aureus/Staphylococcus aureus (8).tif]

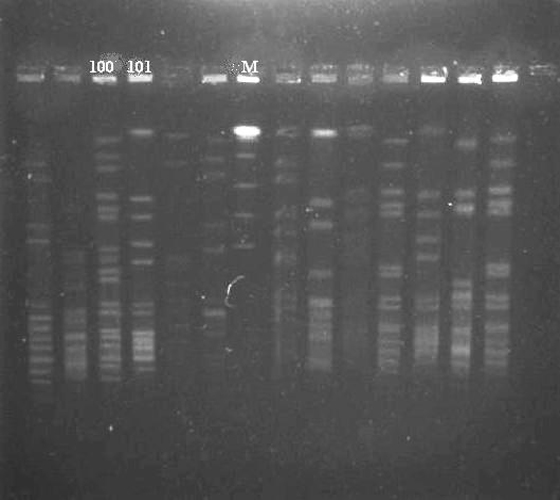

Supplement: Multimedia component 2 [file mmc2.zip › dib_105035_PFGE images for Staphylococcus Aureus/PFGE images for Staphylococcus Aureus/Staphylococcus aureus (9).tif]
